# Incarcerated Rectal Procidentia: A Case Report and Review

**DOI:** 10.7759/cureus.17135

**Published:** 2021-08-12

**Authors:** Abdulqader M Albeladi, Ahmad Odeh, Aminah H AlAli, Abdullah M Alkhars, Kawther Boumarah, Hussain A Al Ghadeer, Sara A Alsaied, Ammar Omrani, Khadir Ahmed

**Affiliations:** 1 General and Laparoscopic Surgery, Prince Saud Bin Jalawy Hospital, Al Ahsa, SAU; 2 General Surgery, Prince Saud Bin Jalawy Hospital, Al Ahsa, SAU; 3 Orthopaedics, King Faisal University, Al Ahsa, SAU; 4 Medicine, King Faisal Univesity, College of Medicine, Dammam, SAU; 5 Paediatrics, Maternity and Children Hospital, Al Ahsa, SAU; 6 General and Colorectal Surgery, Prince Saud Bin Jalawy Hospital, Al Mubarraz, SAU; 7 Laparoscopic Surgery, Prince Saud Bin Jalawy Hospital, Al Mubarraz, SAU; 8 General and Colorectal Surgery, Prince Saud Bin Jalawy Hospital, Al Ahsa, SAU

**Keywords:** rectal procidentia, rectal prolapse, irreducible prolapse, incarcerated prolapse, proctosigmoidectomy

## Abstract

Rectal procidentia is an uncommon perineal disease that is rare in males. There is no specific medical role in treatment of rectal procidentia and surgical intervention is the treatment of choice. Various surgical approaches have been performed, but there is no consensus on which procedure is most effective in terms of patient condition, recurrence rate, bowel function, and risk. This case presentation of a healthy male patient with experience of uncomplicated reducible rectal prolapse and a history of chronic constipation. Presented with complicated rectal prolapse in the presence of incarcerated rectal prolapse after a failed trial with conservative maneuvers, he ended up with abdominal approach sigmoidectomy and posterior mesh rectopexy.

## Introduction

Rectal prolapse is classified according to the level of protrusion into three types: full-thickness (procidentia), partial (mucosal) and internal (intussusception) prolapse [1]. The estimated incidence of rectal prolapse is 2.5 per 100,000. It predominantly affects elderly women with a female:male ratio of 10:1 [2,3]. Rectal procidentia is a pelvic floor disorder in which protrusion of all layers of the rectal wall through the anal verge occurs [4]. The etiology is multifactorial and causes increased intra-abdominal pressure such as constipation or chronic cough, weakness of the anal sphincter, and malnutrition [5]. The diagnosis is established by clinical examination by demonstrating rectal circumferential radial folds with straining maneuvers. Other imaging studies can be used like dynamic pelvic magnetic resonance imaging, manometry, and endoanal ultrasonography [6]. There are two approaches used for treating rectal prolapse: abdominal and perineal approaches [2]. The aim of treatment is to repair prolapse, maintained continence and prevent predisposing factors such as constipation [7].

This case report presents the challenge in management of irreducible massive rectal procidentia, which is rare in males with high recurrence rate, and stresses the challenge of the best management in the emergency department.

## Case presentation

A 50-year-old Saudi male presented to the Emergency Department at Prince Saud Bin Jalawy Hospital, Al Mubarraz, with three days painless anal swelling that started suddenly while straining, irreducible and not associated with abdominal pain. The patient gave history of chronic constipation. He experienced a similar attack 11 years ago that was successfully reduced in hospital. Colonoscopy was done at that time and was normal.

**Figure 1:**
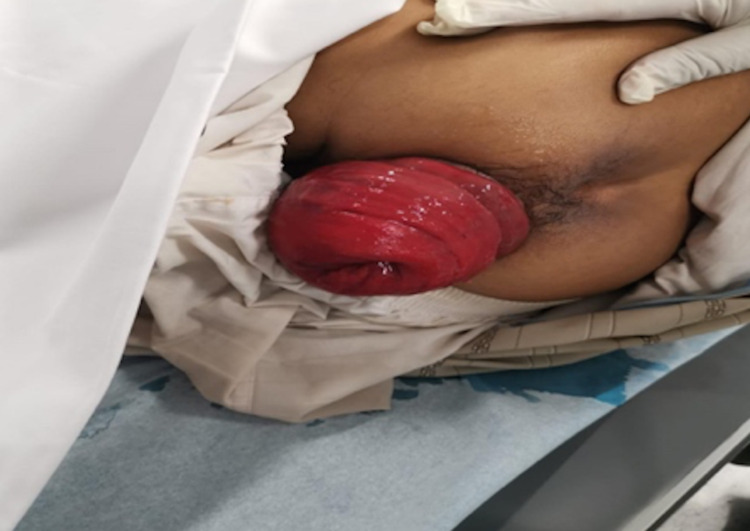
Physical examination showing full thickness of oedematous and congested wall of rectal prolapse

Physical examination revealed full thickness of oedematous and congested wall of rectal prolapse (Figure 1). Several manoeuvres were used to reduce the prolapse: manually, ice packing, sprinkling with sugar and reducing it under spinal anaesthesia but all failed.

The patient underwent exploratory laparotomy, with sigmoidectomy with end to end anastomosis using circular stapler and posterior mesh rectopexy with proline mesh fixed to the sacrum between the posterior rectum and sacral promontory by using absorbable tack and two upper sleeves of mesh that was wrapped around the rectum anteriorly by using proline stitch 2/0 leaving 1 cm space free between them and applied two drains, at the pelvic and over the mesh. Incidental appendectomy was done then the abdomen was closed in layers and skin by stapler.

During hospitalization he was doing fine, vitally stable, tolerating and passing bowel motion. The drainage rate was between 400 - 100 ml/day of hemoserous fluid and was removed. Our patient was discharged on the 11th day of admission in good condition. He was followed up in the general surgery OPD two weeks after discharge, then after one month, and showed no recurrence or reoccurrence of constipation or incontinence. The patient is booked for a colonoscopy for evaluation.

## Discussion

Rectal procidentia is the circumferential protrusion through the anus of all layers of the rectal wall. It should not be confused with haemorrhoids [8]. Full‐thickness rectal prolapse is a distressing condition and can result in serious but rare complications such as gangrene and perforation. Rectal prolapse is most frequently seen in older women. Other risk factors include connective tissue and psychiatric disorders as well as obesity [8-9]. Incarceration and strangulation are the most important complication of rectal prolapse. Often it will reduce spontaneously, at a later stage it requires manual reduction, which with time becomes more frequent and difficult [8-9]. An incarcerated rectal prolapse may be seen after a long history of prolapse as in our case.

Conservative management is the instinctive choice in the emergency department. Conservative methods aim to reduce the edema and allow reduction of the prolapse, with a later planned definitive surgery [8-10]. Edema may be reduced by applying cold compresses to the protruding mass, application of sugar, applying an elastic compression wrap, and placing the patient in the head-down position. Injections in the anal sphincter of local anesthesia can be helpful. After the swelling subsides, an attempt at manual reduction is performed. However, if reduction is not successful or if the prolapse is strangulated, urgent operation is required [9-10].

Demirel and coworkers reported that 20 grams of sugar applied to the prolapse quickly dissolved, and led to reduction of edema and spontaneous reduction [11]. Hovey and Metcalf reported a case in which application of sugar failed to reduce the prolapse; instead the patient developed a perforation [12]. In our case application of sugar failed to help reduce the prolapse. With the absence of proper trials, one must conclude that sugar application is usually unsuccessful. However, surgeons may need to prepare for surgery after a failed trial of sugar application [9-10].

The operative approach to rectal prolapse is controversial. Many factors must be considered, such as the patient's age, gender, comorbidities, and preoperative constipation. Abdominal and perineal operations are the main surgical choices [9].

For elective cases, on a reducible prolapse the choices include the Thiersch stitch, laparotomy and Wells’ rectopexy or resection, and perineal resection. However, laparotomy with resection or a laparotomy with a Wells’ repair have occasionally been carried out in the emergency setting. These operations have low recurrence rates. However, it is difficult to determine the recurrence rate of transabdominal procedures. The other surgical option is perineal rectosigmoidectomy. It can be carried out under spinal anesthesia, and the surgeon avoids a laparotomy. However, perineal rectosigmoidectomy is associated with high recurrence rates. Aziz and Mbembati reported that the procedure of choice in their experience was a perineal proctosigmoidectomy for irreducible prolapse [13]. Our patient underwent exploratory laparotomy, with sigmoidectomy end to end anastomosis using circular stapler and posterior mesh rectopexy of proline mesh fixed to the sacrum.

In the absence of a formal trial, it is difficult to be certain which procedure is the best. Is it the safer perineal proctosigmoidectomy with its high recurrence rate, or the more effective but potentially risky, transabdominal procedures? We suggest that patients with irreducible prolapse should be taken quickly for a surgical procedure, such as an abdominal approach with posterior mesh rectopexy of proline mesh fixed to the sacrum to prevent recurrence.

On the rationale for incisional appendectomy: in otherwise healthy patients aged 10 to 30 years (the group with the highest incidence of appendicitis), incidental appendectomy is an effective means of preventing the morbidity and mortality associated with acute appendicitis. In patients 30 to 50 years of age, incidental appendectomy should be left to the discretion of the surgeon. In patients over 50 years of age, the incidence of acute appendicitis decreases, and the risk associated with surgery and prolonged anesthesia is such that incisional appendectomy is not reasonable. In mentally disabled patients under 50 years of age who are physically healthy, an incidental appendectomy should be performed at the time of other laparotomies. In patients undergoing procedures that may affect access to the appendix in the future, an incidental appendectomy should be performed [14-16]. It is controversial, but the surgeon chooses the best decision for our patient to prevent future morbidity and mortality because the patient is difficult and usually does not ask for medical help.

## Conclusions

Rectal procidentia is an uncommon perineal disease that is rare in males. In general, the prolapsed rectum can be reduced spontaneously; gentle manual reduction and some conservative maneuvers help in reduction like cold compresses of the prolapse, sprinkling with sugar, field block with a local anesthetic, and applying an elastic compression wrap. Untreated rectal prolapse can lead to complications like incarceration and strangulation that need urgent intervention. However, surgical repair is the treatment of choice in the form of two main approaches either abdominal or perineal with considerations in the selection of surgical techniques and favorable outcomes according to patient condition, recurrence rate, bowel function, and risk. Finally, we suggest that patients with irreducible prolapse should undergo an abdominal approach with posterior mesh rectopexy of proline mesh.
